# Infertility Caused by Pelvic Tuberculosis: A Forgotten and Rare Diagnosis

**DOI:** 10.1155/crdi/3837614

**Published:** 2026-05-25

**Authors:** Maryam Hashemi, Maryam Dehghan

**Affiliations:** ^1^ Department of Minimally Invasive Surgery Gynecology, School of Medicine, Al-Zahra Hospital, Isfahan University of Medical Sciences, Isfahan, Iran, mui.ac.ir

**Keywords:** infertility, pregnancy, tuberculosis

## Abstract

**Introduction:**

Active tuberculosis (TB), caused by the *Mycobacterium TB* bacillus, has the highest mortality rate among all infectious diseases worldwide. TB can be classified into two forms: pulmonary and extrapulmonary. The majority of patients infected with TB clear the mycobacterium after the primary infection. Genital TB is usually secondary to hematogenous spread from a primary site, typically the lungs. Fertility can be impaired by genital TB, primarily due to involvement of the fallopian tubes, which are the most commonly affected site. In addition, infection of the uterus, ovaries, and peritoneum has also been reported.

**Case Presentation:**

We describe two cases of pelvic TB identified during infertility workups focused on fallopian tube involvement. Both cases were managed with a combination of surgical and medical therapies.

**Discussion:**

Female genital TB typically presents with infertility, abdominal pain, or menstrual disturbances. Imaging studies often reveal nonspecific findings. Laparoscopic surgery can provide more definitive diagnostic information. A biopsy is useful for confirming the diagnosis of TB more accurately and rapidly. Treatment of genital TB requires a four‐drug regimen due to the high recurrence rate and the prevalence of drug‐resistant strains. In cases of large abscesses or fallopian tube obstruction, surgical intervention may be necessary.

**Conclusion:**

Given the increasing trends in immigration from TB‐endemic areas and the established relationship between pelvic TB and infertility, it is advisable to consider TB as a potential cause of infertility in women from these regions.

## 1. Introduction

Active tuberculosis (TB), caused by *Mycobacterium TB*, has the highest mortality rate among all infectious diseases worldwide. The majority of cases occur in low‐ and middle‐income countries. Despite various preventive strategies, more than 10 million people continue to develop TB each year [1].

TB can be classified into two forms: pulmonary and extrapulmonary. Extrapulmonary TB affects various sites, including the kidneys, bones, central nervous system, gastrointestinal tract, female genital tract, and peritoneum. It accounts for 15%–20% of all TB cases; however, female pelvic TB is rare, representing only about 5% of extrapulmonary cases [2]. TB is primarily transmitted through airborne particles, with lung involvement occurring in 79%–87% of active TB infections. Most patients clear the *Mycobacterium* after the primary infection. In 5%–10% of cases, the disease enters a latent phase, with reactivation occurring later in life [3]. Extrapulmonary TB may develop following progressive primary disease or reactivation of latent infection [4].

Genital TB is typically secondary to hematogenous spread from a primary site, most commonly the lungs. Fertility can be compromised by genital TB, primarily due to involvement of the fallopian tubes, which are the most frequently affected sites. This involvement can lead to hydrosalpinx or pyosalpinx and dense adhesions, resulting in subfertility and an increased risk of ectopic pregnancy. In addition, infection of the uterus, ovaries, and peritoneum has been documented [5, 6]. TB can also increase morbidity and mortality rates if pregnancy occurs [7]. Here, we report two cases of pelvic TB diagnosed during infertility workup.

### 1.1. Case 1

An 18‐year‐old nulligravid woman with a 3‐year history of primary infertility, who recently immigrated from a TB‐endemic area, was referred to our clinic. The purified protein derivative (PPD) test was positive, and her hysterosalpingography revealed obstruction of both tubes (Figure 1(a)). She has a history of pulmonary TB 6 years ago that was not treated. Diagnostic laparoscopy revealed disseminated nodular peritoneal lesions suggestive of TB, bilateral fallopian tube phimosis with caseous granulomas, ovarian adhesions, and severe Fitz–Hugh–Curtis syndrome (FHCS) (Figures 1(b), 1(c), and 1(d)). Considering the normal appearance of the uterine cavity on hysterosalpingography and the normal menstrual pattern, Asherman’s syndrome was not suspected, and hysteroscopy was not performed. Adhesiolysis and peritoneal biopsy were performed, and peritoneal TB was confirmed based on positive polymerase chain reaction (PCR) and acid‐fast bacteria (AFB) culture results. Consequently, a four‐drug regimen consisting of isoniazid, rifampin, pyrazinamide, and ethambutol was prescribed for 6 months.

FIGURE 1Bilateral fallopian tube obstruction observed in hysterosalpingography (a); tubercles (yellow arrows) and caseous granulomas (white arrows), along with right tube phimosis (blue arrows) (b); right tube phimosis (blue arrows) (c); and Fitz–Hugh–Curtis syndrome (d).

**Figure:**
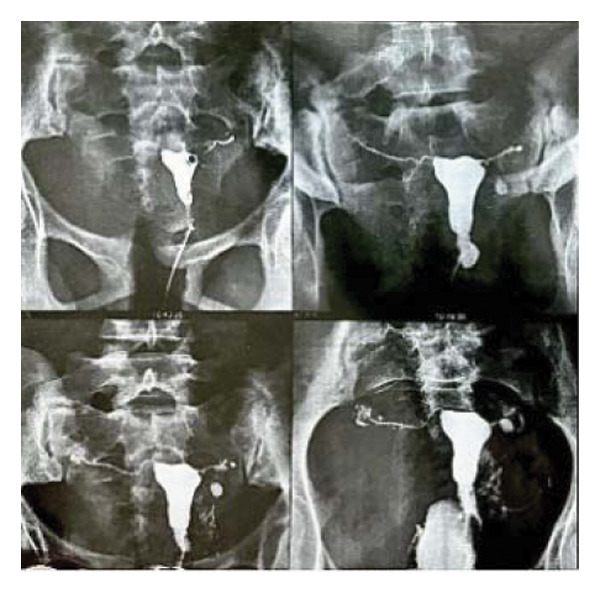
(a)

**Figure:**
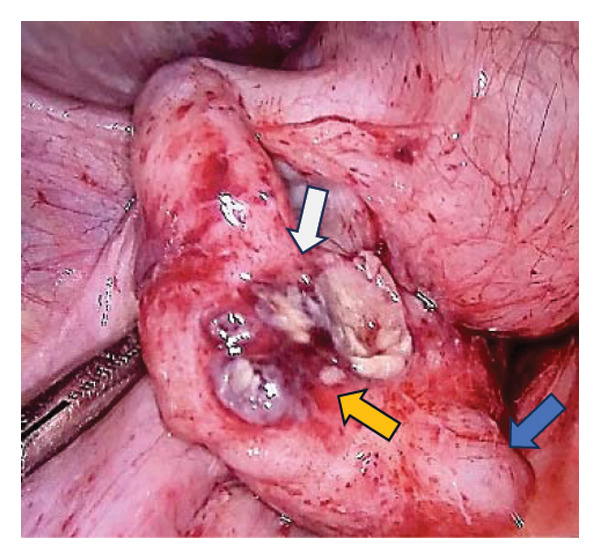
(b)

**Figure:**
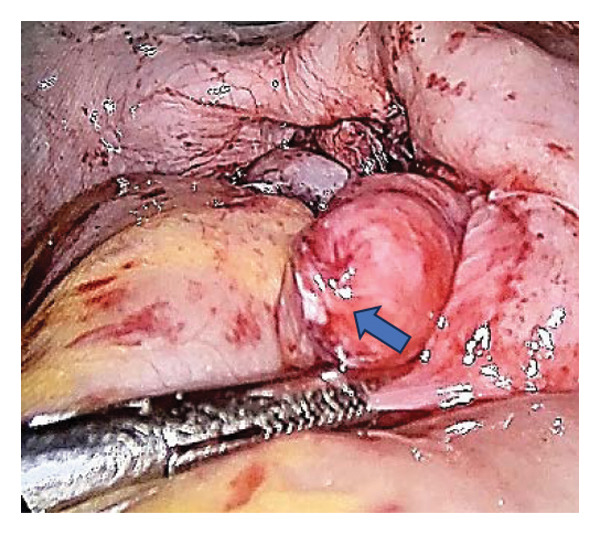
(c)

**Figure:**
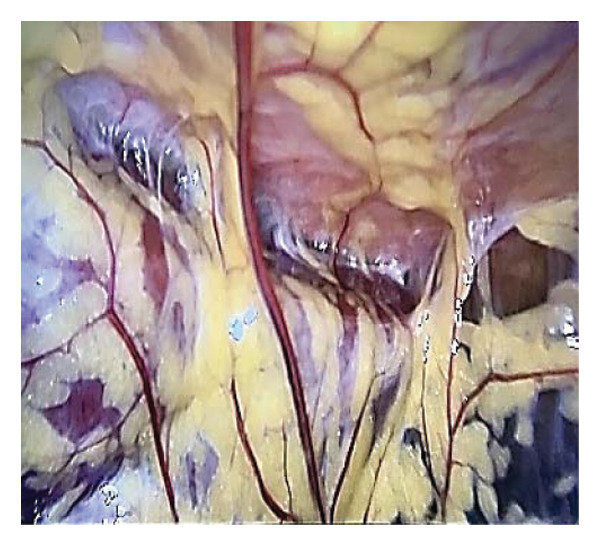
(d)

### 1.2. Case 2

A 21‐year‐old nulligravid woman with a 5‐year history of primary infertility and a normal menstrual pattern, who recently immigrated from a TB‐endemic area, was referred to our clinic. The PPD test was positive, and her hysterosalpingography revealed obstruction of the right fallopian tube and hydrosalpinx of the left fallopian tube (Figure 2(a)). She has no history of pulmonary TB. Diagnostic laparoscopy revealed disseminated peritoneal nodular lesions suggestive of TB, ovarian adhesions, and FHCS. Although in this patient, the fallopian tube appearance was normal, a granuloma caseous in the right ovarian fossa was seen (Figures 2(b), 2(c), and 2(d)). In this case, dye injection demonstrated that both fallopian tubes were patent. Adhesiolysis and peritoneal biopsy were performed, and peritoneal TB was confirmed based on positive PCR and AFB culture results. Consequently, a four‐drug regimen consisting of isoniazid, rifampin, pyrazinamide, and ethambutol was prescribed for 6 months in this patient, as well.

FIGURE 2Right fallopian tube obstruction and left fallopian tube hydrosalpinx observed in hysterosalpingography (a); peritoneal tubercles indicated by yellow arrows (b); caseous granuloma shown by white arrows in the left ovarian fossa (c); and Fitz–Hugh–Curtis syndrome (d).

**Figure:**
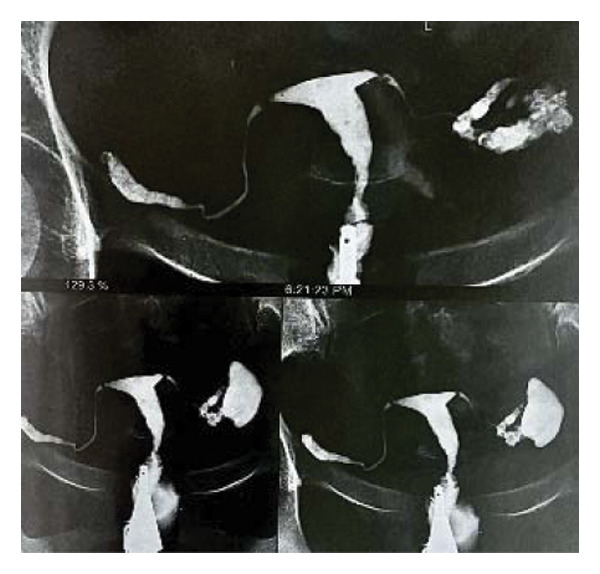
(a)

**Figure:**
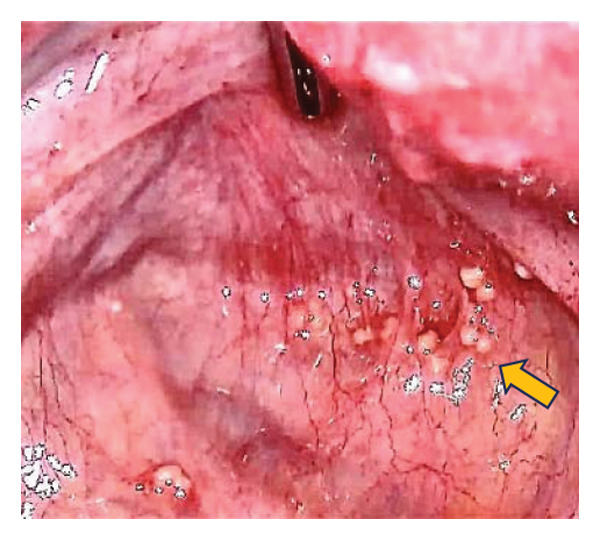
(b)

**Figure:**
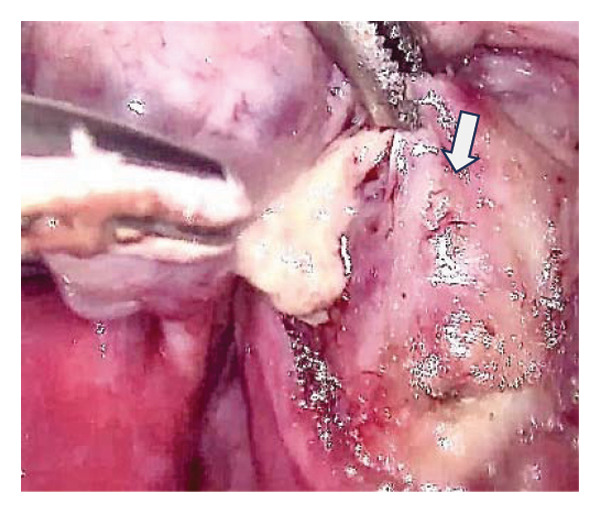
(c)

**Figure:**
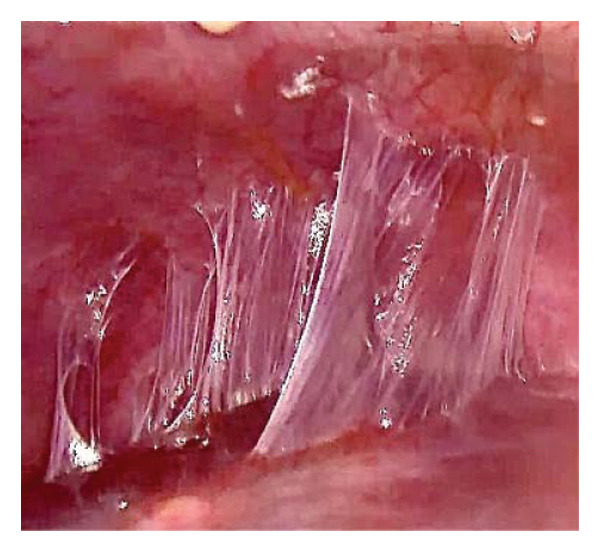
(d)

### 1.3. Follow‐Up

Since our patients were unwilling to use ART and considering their young age, adequate ovarian reserve, and acceptable Endometriosis Fertility Index (EFI), the decision—made by the patients and the medical team after completing the course of antibiotic treatment and consulting with the couples—was to allow an opportunity for spontaneous pregnancy.

## 2. Discussion

We report two cases of pelvic TB identified during infertility evaluations, both involving fallopian tube pathology. Each case was managed with a combination of surgical intervention and medical therapy. Neither patient exhibited pulmonary symptoms at the time of referral; the only factor prompting suspicion was their recent migration from TB‐endemic regions.

Female genital TB most commonly presents with infertility but can also manifest as abdominal pain or menstrual disturbances [8]. In a case series by Kaya et al., involving five women diagnosed with genital TB, all presented with infertility, while pelvic pain and irregular vaginal bleeding were each reported in only one case [9]. Imaging often reveals nonspecific signs such as fallopian tube dilatation, strictures, nodular scarring, and hydrosalpinx. When the endometrium is involved, ultrasound may show a dilated endometrium containing heterogeneous material or calcifications [8]. Laparoscopic surgery can identify more specific findings, such as tubercles or caseous granulomas in the fallopian tubes or peritoneum. Biopsy of these lesions, combined with interferon‐gamma release assays (IGRAs) or microbial tests such as PCR, can provide a more accurate and rapid diagnosis of TB [10]. Data from 32 patients with genital TB in a recent systematic review spanning a 21‐year observational period (2000–2021) revealed that the primary histologic finding was the presence of epithelioid cell granulomas, observed in 81.25% of patients. Langhans‐type multinucleated giant cells were present in 46.87% of cases. AFB was detected in tissue sections in 46.87% of cases after Ziehl–Neelsen (ZN) staining. Seven patients (21.87%) had positive PCR results [11]. In our patients, tubercles or caseous granulomas were observed in the fallopian tubes and peritoneum, and biopsy confirmed the diagnosis of TB.

Genital TB should be treated with a four‐drug regimen due to the high recurrence rate and the prevalence of drug‐resistant TB strains. This regimen typically includes a combination of antibacterial drugs—isoniazid, rifampicin, pyrazinamide, and streptomycin—and bacteriostatic agents such as ethambutol or ethionamide, administered for 9–12 months depending on the resistance pattern [1, 4]. In cases involving large abscesses or fallopian tube obstruction, surgical intervention may be necessary. Although successful pregnancies have been reported following medical therapy for endometrial TB, surgery may be required when fallopian tube obstruction is present [7]. Jindal et al. found that among women with silent endometrial TB, antitubercular therapy (ATT) improved the likelihood of spontaneous pregnancy. They reported that over 90% of women conceived within the first 12 months—either during ATT administration or within 6 months after treatment completion. However, in cases of severe damage, especially involving the fallopian tubes, spontaneous pregnancy is unlikely and assisted reproductive technologies (ARTs) should be used. Although the chances of success of these methods are also limited, they can be considered [12]. In a study of seven cases of genital TB with tubal involvement, only 2 out of 7 patients (28.6%) were able to conceive through ART [13]. We managed our cases with a combination of surgery and ATT; however, given the lack of successful conception, ART should be considered.

Although screening for TB in all women with impaired fertility or during first‐trimester screening is generally not beneficial, some experts recommend screening during preconception or first‐trimester evaluations for women with risk factors. These risk factors include immigration from TB high‐endemic regions, HIV infection, or unexplained subfertility, especially given the rising immigration trends and the slowing decline in TB incidence. The QuantiFERON‐TB test is the preferred screening method. If the test is positive, further evaluation with biopsy or PCR sampling from the fallopian tubes or endometrium may be performed [7].

## 3. Conclusion

Given the increasing trends in immigration from TB‐endemic areas and the well‐established association between pelvic TB, infertility, and fetomaternal morbidity and mortality, it is advisable to consider TB as a potential cause of infertility in women from endemic regions. Early diagnosis is crucial to prevent further complications.

## Author Contributions

Maryam Hashemi and Maryam Dehghan both contributed to patient management and manuscript writing.

## Funding

The authors received no financial support for the research, authorship, or publication of this article.

## Ethics Statement

This study was approved by the Research Committee of Isfahan University of Medical Sciences.

## Consent

Written informed consent was obtained from the patient for the publication of this case report and any accompanying images.

## Conflicts of Interest

The authors declare no conflicts of interest.

## Data Availability

The data that support the findings of this study are available from the corresponding author upon reasonable request.
